# South West Orthopaedic Club

**Published:** 1984-04

**Authors:** 


### South West Orthopaedic Club

### Meeting at Yeovil, 19 December 1983

### SOME PELVIC TUMOURS

Mr. A. H. C. Ratliff, Bristol Royal Infirmary

Mr. Ratliff presented a series of patients who had been treated for tumours of the pelvis and in particular in the region of the acetabulum. These included patients with an eosinophilic granuloma, an aneurysmal bone cyst and several with chondrosarcomata. Treatment of these patients was described, two of which eventually had hind quarter amputation. There was lively discussion following this paper.

H.R.

### THE USE OF ENDER'S NAILS IN UNSTABLE TROCHANTERIC FEMORAL FRACTURES

C. H. Marsh
Bath and Wessex Orthopaedic Hospital

Eighty consecutive patients with unstable trochanteric femoral fractures were reviewed following treatment by the Ender technique. Fracture stability was assessed using Jensens modification of the Evans classification, as this allows good prediction of the risk of post-fixation instability and redislocation of the fragments. 60% of the operative procedures were performed by senior House Officer grade surgeons. The rate of general complications was similar to that reported in previous studies but the mortality rate was high, 28% by 6 weeks. Early fixation failure occurred in 18% of patients and needed a revision procedure to restore mobility. Leg shortening and external rotation deformities due to uncontrolled fragmentary collapse was found in almost 80% of the 57 surviving patients at 3 months, and caused severe disability. The Ender technique allows no opportunity to directly manipulate unstable fractures into the optimum position which allows collapse into a stable position of bony impaction and the implant controls such collapse poorly. These factors are felt to be responsible for the unacceptably high rates of limb deformity seen in this series. The continued use of Ender's nails in unstable fractures is questioned.

### VIBRATION ANALYSIS IN THE ASSESSMENT OF FRACTURE HEALING

W. J. Mintowt-Czyz, J. A. Fairclough, I. Mackie and L. Nokes, Prince of Wales Orthopaedic Hospital, Cardiff

When an object is made to vibrate it does so at a frequency which is determined by the physical characteristics of that object. If the integrity of the vibrating system is disrupted then it will vibrate at a different frequency or series of separate frequencies depending on the nature of the disruption. By driving the human tibia across a spectrum of frequencies, and monitoring the resultant vibration of the bone from skin mounted accelerometers, the natural frequency of the underlying bone can be determined whether it be intact or fractured. The changes in natural frequency of tibial fracture fragments were measured during healing in eight patients and were found to change characteristically as healing occurred.

A second technique which depends on measuring the attenuation of vibration as it is transmitted along bone was also described. The response of the tibia to an impulse applied to the tibial tubercle was investigated by measuring the amplitude of vibration at each end of the bone using skin mounted accelerometers. In the presence of a fracture transmission of vibration is markedly attenuated, but as healing progresses that attenuation becomes less with time, and approaches normal levels as the fracture consolidates. Data was collected from 32 cases of tibial fractures that went on to satisfactory union. Two cases of non-union were also studied.

The healing curves derived from both techniques are closely similar both in their exponential shape and the timescale over which the changes occur. The rate of fracture healing was inferred from the slopes of the two healing curves, while the extent of change on the y axis of the curves was seen as a measure of the degree of fracture healing. In two cases of non-union the fractured bones yielded results significantly different from those got from normally healing bones, and both the rate and extent of healing were shown to be deficient.

It was concluded that these two techniques might allow the quantitative and objective assessment of both the rate and extent of fracture healing, and might offer the hope of early diagnosis of delayed and non-union.

### LEAKAGE UNDER THE TOURNIQUET DURING BIER'S BLOCKADE

**P. Hutton, M. M. El Hassan and A. M. S. Black, Department of Anaesthesia, Bristol Royal Infirmary**

Recently there has been considerable critcism in the public press about the use of Bier's blockade. This was also the subject of a leading article in 1980 in the B.M.J. Problems, when they occur, are due to high doses of local anaesthetic escaping into the general circulation causing convulsions and cardio-respiratory arrest. Whilst some documented cases are undoubtedly due to poor technique, others are not. In the latter, the method of leakage of local anaesthetic has remained a mystery, a common assumption being that it travels through interosseous channels in the bone which are unaffected by tourniquet pressure.

An 18 year old girl convulsed in our casualty department during intravenous regional anaesthesia and the reduction of her left wrist fracture was abandoned. She presented the next day for a formal reduction under general anaesthesia and consented to an investigation intended to simulate the previous day's incident. First, general anaesthesia was established, and after an unsuccessful attempt to re-cannulate the left median cubital vein (which had been used the previous day) a cannula was inserted into the left lateral cubital vein. The arm was drained by gravity for two minutes, a tourniquet was inflated at midtriceps level to 300 mmHg and 200 µg of fentanyl diluted in 40 mls of normal saline was injected over one minute. The tourniquet was kept inflated for six minutes during which time blood samples were taken from the opposite cubital fossa. Subsequent analysis demonstrated that fentanyl had passed into the systemic circulation during inflation of the cuff. This finding prompted a study of the venous pressures generated during such a procedure.

In three volunteers the pressure inside the median cubital vein (distal to an inflated tourniquet) was measured during and after the injection of 40 mls of 0.9% saline through a separate cannula situated either in the cubital fossa vein or in a vein on the back of the hand. For different injection rates the tourniquet inflation pressure was varied over the range 100–400 mmHg. The veins in the upper arm which were compressed by the tourniquet appeared to act as 'Starling' type resistors. The maximal pressure obtained from the injections increased with increasing tourniquet pressure, but never exceeded it by more than 10 mmHg. In one subject an intra-luminal venous pressure of over 400 mmHg could easily be obtained if the tourniquet pressure was similarly high.

The attainment of a maximal sustained 'plateau' pressure limited by tourniquet pressure indicated leakage of injectate under the tourniquet. This was confirmed by including 10 mg of indocyanine green in the 40 mls of injectate, and measuring the concentration in venous blood sampled from the other arm whilst the tourniquet was still inflated. Whether or not leakage under the cuff occurred depended upon the individual subject, and the site and speed of injection.

Injections in the ante cubital fossa could achieve leakage conditions at a much slower rate of injection than those given into a vein on the back of the hand.

The dangers of cubital fossa injection for Bier's blockade needs further emphasis. This is particularly relevant to less experienced medical personnel who may have difficulty in cannulating a peripheral vein and be tempted to use larger cubital fossa veins for the injection of local anaesthetic.

